# TH1 and TH2 cytokines dataset in insulin users with diabetes mellitus and newly diagnosed breast cancer

**DOI:** 10.1016/j.dib.2017.02.028

**Published:** 2017-02-20

**Authors:** Zachary A.P. Wintrob, Jeffrey P. Hammel, George K. Nimako, Dan P. Gaile, Alan Forrest, Alice C. Ceacareanu

**Affiliations:** a State University of New York at Buffalo, Department of Pharmacy Practice, NYS Center of Excellence in Bioinformatics and Life Sciences, 701 Ellicott Street, Buffalo, NY 14203, USA; b Cleveland Clinic, Dept. of Biostatistics and Epidemiology, 9500 Euclid Ave., Cleveland, OH 44195, USA; c State University of New York at Buffalo, Department of Biostatistics, 718 Kimball Tower, Buffalo, NY 14214, USA; d The UNC Eshelman School of Pharmacy, Division of Pharmacotherapy and Experimental Therapeutics, Campus Box 7569, Chapel Hill, NC 27599, USA; e Roswell Park Cancer Institute, Dept. of Pharmacy Services, Elm & Carlton Streets, Buffalo, NY 14263, USA

**Keywords:** Type 1 T helper cytokine, Type 2 T helper cytokine, TH1 cytokine, TH2 cytokine, Cytokine, Insulin, Breast cancer, Diabetes, Cancer outcomes, Cancer prognosis

## Abstract

Exogenous insulin use may interfere with the T helper cells’ cytokine production. This dataset presents the relationship between pre-existing use of injectable insulin in women diagnosed with breast cancer and type 2 diabetes mellitus, the T-helper 1 and 2 produced cytokine profiles at the time of breast cancer diagnosis, and subsequent cancer outcomes. A Pearson correlation analysis evaluating the relationship between T-helper cytokines stratified by of insulin use and controls is also provided.

**Specifications Table**

**Table:** 

Subject area	Clinical and Translational Research
More specific subject area	Biomarker Research, Cancer Epidemiology
Type of data	Tables
How data was acquired	Tumor registry query was followed by vital status ascertainment, and medical records review
	Luminex®-based quantitation from plasma samples was conducted for the following T-helper 1 and T-helper 2 cytokines: Interleukine-2, soluble interleukine-2 receptor α, interleukine-12 subunit p40, interleukine-12 subunit p70, interferon α 2, interferon γ, chemokine ligand 10 (interferon gamma-induced protein 10), chemokine ligand 9 (monokine-induced by interferon γ), chemokine ligand 8 (interleukine-8) interleukine-5, interleukine-10, and interleukine-13.
	A Luminex®200^TM^ instrument with Xponent 3.1 software was used to acquire all data.
Data format	Analyzed
Experimental factors	T-helper 1 and 2 produced cytokines were determined from the corresponding plasma samples collected at the time of breast cancer diagnosis
Experimental features	The dataset included 97 adult females with diabetes mellitus and newly diagnosed breast cancer (cases) and 194 matched controls (breast cancer only). Clinical and treatment history were evaluated in relationship with cancer outcomes and factor-helper 1 and 2 produced cytokine profiles. A biomarker correlation analysis was also performed.
Data source location	United States, Buffalo, NY - 42° 53′ 50.3592′′N; 78° 52′ 2.658′′W
Data accessibility	The data is with this article

**Value of the data**

• This dataset represents the observed relationship between administration of exogenous insulin, circulating T-helper 1 and 2 produced cytokines at breast cancer diagnosis and cancer outcomes.
• The data we present here has the potential to guide future research evaluating the role of insulin in the modulation of type 1 and type 2 immunity.
• Our observations can assist further research exploring the relationship between insulin administration and T-helper-driven signaling in breast cancer occurrence.

## 1 Data

Reported data represents the observed association between pre-existing use of injectable insulin before breast cancer diagnosis and the T-helper 1 and 2 produced cytokine profiles upon cancer diagnosis in women with both breast cancer and diabetes mellitus (Table 1). Data in Table 2 includes the observed correlations between T-helper 1 and 2 cytokines stratified by diabetes mellitus pharmacotherapy and controls.

## 2 Experimental design, materials and methods

Evaluation of the association between profiles of T-helper 1 and 2 produced cytokines, injectable insulin use and BC outcomes was carried out under two protocols approved by both Roswell Park Cancer Institute (EDR154409 and NHR009010) and the State University of New York at Buffalo (PHP0840409E). Demographic and clinical patient information was linked with cancer outcomes and profiles of T-helper 1 and 2 produced cytokines of corresponding plasma specimen harvested at BC diagnosis and banked in the Roswell Park Cancer Institute Data Bank and Bio-Repository.

### 2.1 Study population

All incident breast cancer cases diagnosed at Roswell Park Cancer Institute (01/01/2003-12/31/2009) were considered for inclusion (*n*=2194). Medical and pharmacotherapy history were used to determine the baseline presence of diabetes.

### 2.2 Inclusion and exclusion criteria

All adult women with pre-existing diabetes at breast cancer diagnosis having available banked treatment-naïve plasma specimens (blood collected prior to initiation of any cancer-related therapy - surgery, radiation or pharmacotherapy) in the Institute’s Data Bank and Bio-Repository were included.

Subjects were excluded if they had prior cancer history or unclear date of diagnosis, incomplete clinical records, type 1 or unclear diabetes status. For a specific breakdown of excluded subjects, please see the original research article by Wintrob et al. [1].

A total of 97 female subjects with breast cancer and baseline diabetes mellitus were eligible for inclusion in this analysis.

### 2.3 Control-matching approach

Each of the 97 adult female subjects with breast cancer and diabetes mellitus (defined as “cases”) was matched with two other female subjects diagnosed with breast cancer, but without baseline diabetes mellitus (defined as “controls”). The following matching criteria were used: age at diagnosis, body mass index category, ethnicity, menopausal status and tumor stage (as per the American Joint Committee on Cancer). Some matching limitations applied [1].

### 2.4 Demographic and clinical data collection

Clinical and treatment history was documented as previously described [1]. Briefly, users of any insulin were defined as patients receiving a form of injectable insulin – alone or in combination – at the time of breast cancer diagnosis. Vital status was obtained from the Institute’s Tumor Registry, a database updated biannually with data obtained from the National Comprehensive Cancer Networks’ Oncology Outcomes Database. Outcomes of interest were breast cancer recurrence and/or death. Details regarding patient demographics and clinical characteristics have been previously published [1].

### 2.5 Plasma specimen storage and retrieval

All the plasma specimens retrieved from long-term storage were individually aliquoted in color coded vials labeled with unique, subject specific barcodes. Overall duration of freezing time was accounted for all matched controls ensuring that the case and matched control specimens had similar overall storage conditions. Only two instances of freeze-thaw were allowed between biobank retrieval and biomarker analyses: aliquoting procedure step and actual assay.

### 2.6 Luminex® assays

A total of 12 biomarkers - interleukine-2, soluble interleukine-2 receptor α, interleukine-12 subunit p40, interleukine-12 subunit p70, interferon α 2, interferon γ, chemokine ligand 10 (interferon gamma-induced protein 10), chemokine ligand 9 (monokine-induced by interferon γ), chemokine ligand 8 (interleukine-8), interleukine-5, interleukine-10, and interleukine-13 - were quantified according to the manufacturer protocol. The Luminex® HCYTOMAG-60K panel (Millipore Corporation, Billerica, MA) was used in this study.

### 2.7 Biomarker-pharmacotherapy association analysis

Biomarker cut-point optimization was performed for each analyzed biomarker. Biomarker levels constituted the continuous independent variable that was subdivided into two groups that optimized the log rank test among all possible cut-point selections yielding a minimum of 10 patients in any resulting group. Quartiles were also constructed. The resultant biomarker categories were then tested for association with type 2 diabetes mellitus therapy and controls by Fisher’s exact test. The continuous biomarker levels were also tested for association with diabetes therapy and controls across groups by the Kruskal-Wallis test and pairwise by the Wilcoxon rank sum. Multivariate adjustments were performed accounting for age, tumor stage, body mass index, estrogen receptor status, and cumulative comorbidity. The biomarker analysis was performed using R Version 2.15.3. Please see the original article for an illustration of the analysis workflow [1].

Correlations between biomarkers stratified by type 2 diabetes mellitus pharmacotherapy and controls were assessed by the Pearson method. Correlation models were constructed both with and without adjustment for age, body mass index, and the combined comorbidity index. Correlation analyses were performed using SAS Version 9.4.

## Funding sources

This research was funded by the following grant awards: Wadsworth Foundation Peter Rowley Breast Cancer Grant awarded to A.C.C. (UB Grant number 55705, Contract CO26588).

## Figures and Tables

**Table 1:** T-helper 1 and 2 produced cytokines׳ associations with insulin use.

Biomarker	Biomarker Grouping	Concentration (ng/ml)	Control	No Insulin	Any Insulin	Unadjusted p-value (MVP)			
						p^1^	p^2^	p^3^	Global Test
IL-2 (pg/ml)	Median	–	1.60	1.60	1.60	0.680	0.970	0.860	0.920
	(25th–75th)		(1.60–3.2)	(1.60–3.2)	(1.60–3.66)	(0.350)	(0.470)	(0.800)	(0.410)
	OS-Based	0.10–34.18	189 (97.4%)	72 (94.7%)	19 (95.0%)	0.270	0.450	1.000	0.320
	Optimization	**35.37–516.64**	**5 (2.6%)**	**4 (5.3%)**	**1 (5.0%)**	(0.200)	(0.450)	(0.590)	(0.360)
	DFS-Based	**0.10–1.94**	**131 (67.5%)**	**49 (64.5%)**	**13 (65.0%)**	0.630	0.820	0.970	0.880
	Optimization	1.99–516.64	63 (32.5%)	27 (35.5%)	7 (35.0%)	(0.560)	(0.640)	(0.880)	(0.700)
									
sIL-2Rα (pg/ml)	Median	–	3.20	7.06	49.52	0.490	0.048	0.140	0.130
	(25th–75th)		(1.60–47.32)	(1.60–60.70)	(1.68–117.66)	(0.320)	(0.110)	(0.360)	(0.220)
	Quartiles	0.00–1.60	84 (43.3%)	30 (39.5%)	5 (25.0%)	0.820	0.140	0.450	0.440
		1.70–7.00	16 (8.2%)	8 (10.5%)	2 (10.0%)				
		7.12–57.42	50 (25.8%)	18 (23.7%)	4 (20.0%)				
		57.68 to ALQ	44 (22.7%)	20 (26.3%)	9 (45.0%)				
	OS-Based	0.00–63.34	155 (79.9%)	57 (75.0%)	11 (55.0%)	0.380	0.021	0.090	0.044
	Optimization	**63.37 to ALQ**^a^	**39 (20.1%)**	**19 (25.0%)**	**9 (45.0%)**	(0.130)	(0.012)	(0.070)	(0.021)
	DFS-Based	0.00–62.50	153 (78.9%)	57 (75.0%)	11 (55.0%)	0.490	0.025	0.090	0.060
	Optimization	**62.65 to ALQ**^a^	**41 (21.1%)**	**19 (25.0%)**	**9 (45.0%)**	(0.210)	(0.018)	(0.070)	(0.033)
									
IL-12p40 (pg/ml)	Median	–	8.16	12.67	11.43	0.170	0.380	0.920	0.320
	(25th–75th)		(1.75–30.81)	(3.20–30.78)	(5.43–24.29)	(0.260)	(0.280)	(0.990)	(0.370)
	Quartiles	1.25–3.20	74 (38.1%)	20 (26.3%)	4 (20.0%)	0.210	0.230	0.620	0.230
		3.94–9.74	29 (14.9%)	13 (17.1%)	6 (30.0%)				
		9.94–30.67	42 (21.6%)	24 (31.6%)	5 (25.0%)				
		30.92–2045.71	49 (25.3%)	19 (25.0%)	5 (25.0%)				
	OS-Based	1.25–3.12	53 (27.3%)	14 (18.4%)	3 (15.0%)	0.130	0.240	1.000	0.200
	Optimization	**3.20–2045.71**	**141 (72.7%)**	**62 (81.6%)**	**17 (85.0%)**	(0.260)	(0.190)	(0.890)	(0.330)
	DFS-Based	1.25–3.12	53 (27.3%)	14 (18.4%)	3 (15.0%)	0.130	0.240	1.000	0.200
	Optimization	**3.20–2045.71**^a^	**141 (72.7%)**	**62 (81.6%)**	**17 (85.0%)**	(0.260)	(0.190)	(0.890)	(0.330)
									
IL-12p70 (pg/ml)	Median	–	1.60	2.66	2.81	0.070	0.260	0.850	0.130
	(25th–75th)		(1.60–3.20)	(1.60–4.64)	(1.60–6.85)	(0.080)	(0.830)	(0.660)	(0.190)
	OS-Based	**0.10–0.59**	**5 (2.6%)**	**3 (3.9%)**	**3 (15.0%)**	0.690	0.029	0.100	0.041
	Optimization	0.70–2510.07	189 (97.4%)	73 (96.1%)	17 (85.0%)	(0.580)	(0.080)	(0.090)	(0.120)
	DFS-Based	0.10–2.20	120 (61.9%)	36 (47.4%)	9 (45.0%)	0.031	0.150	0.850	0.052
	Optimization	**2.28–2510.07**	**74 (38.1%)**	**40 (52.6%)**	**11 (55.0%)**	(0.090)	(0.260)	(0.900)	(0.160)
									
IFN-α2 (pg/ml)	Median	–	7.24	6.74	12.49	0.610	0.032	0.110	0.110
	(25th–75th)		(3.20–13.61)	(3.20–17.55)	(7.32–18.70)	(0.580)	(0.210)	(0.410)	(0.360)
	Quartiles	0.61–1.60	56 (28.9%)	24 (31.6%)	2 (10.0%)	0.370	0.220	0.130	0.240
		3.47–7.40	42 (21.6%)	17 (22.4%)	4 (20.0%)				
		7.43–15.15	52 (26.8%)	13 (17.1%)	7 (35.0%)				
		15.32–1880.18	44 (22.7%)	22 (28.9%)	7 (35.0%)				
	OS-Based	0.61–4.18	63 (32.5%)	26 (34.2%)	3 (15.0%)	0.780	0.120	0.110	
	Optimization	**4.18–1880.18**^a^	**131 (67.5%)**	**50 (65.8%)**	**17 (85.0%)**	(0.430)	(0.130)	(0.110)	(0.200)
	DFS-Based	0.61–2.66	29 (14.9%)	8 (10.5%)	1 (5.0%)	0.340	0.320	0.680	0.400
	Optimization	**2.93–1880.18**	**165 (85.1%)**	**68 (89.5%)**	**19 (95.0%)**	(0.140)	(0.100)	(0.530)	(0.150)
									
IFN-γ (pg/ml)	Median	–	13.32	11.43	7.59	0.230	0.670	0.710	0.460
	(25th–75th)		(4.70–36.30)	(2.55–41.45)	(5.21–48.19)	(0.800)	(0.790)	(0.700)	(0.970)
	Quartiles	0.07–3.86	42 (21.6%)	28 (36.8%)	3 (15.0%)	0.026	0.030	0.008	0.004
		4.03–12.43	50 (25.8%)	12 (15.8%)	10 (50.0%)				
		12.55–37.33	56 (28.9%)	15 (19.7%)	1 (5.0%)				
		38.74–646.43	46 (23.7%)	21 (27.6%)	6 (30.0%)				
	OS-Based	0.07–230.77	188 (96.9%)	72 (94.7%)	20 (100%)	0.470	1.000	0.580	0.540
	Optimization	**376.09–646.43**	**6 (3.1%)**	**4 (5.3%)**	**0 (0%)**	(0.370)	(0.320)	(0.120)	(0.310)
	DFS-Based	0.07–187.14	187 (96.4%)	72 (94.7%)	19 (95.0%)	0.510	0.550	1.000	0.610
	Optimization	**206.34–646.43**^a^	**7 (3.6%)**	**4 (5.3%)**	**1 (5.0%)**	(0.560)	(0.760)	(0.770)	(0.820)
									
CXCL-10 (IP-10, pg/ml)	Median	–	488	463	564	0.610	0.870	0.930	0.880
	(25th–75th)		(347–814)	(338–688)	(352–733)	(0.690)	(0.260)	(0.300)	(0.420)
	Quartiles	1.6–344.8	48 (24.7%)	20 (26.3%)	5 (25.0%)	0.970	0.780	0.800	0.950
		346.1–484.3	48 (24.7%)	20 (26.3%)	4 (20.0%)				
		484.5–748.4	47 (24.2%)	18 (23.7%)	7 (35.0%)				
		751.0–3745.0	51 (26.3%)	18 (23.7%)	4 (20.0%)				
	OS-Based	1.6–428.3	81 (41.8%)	33 (43.4%)	9 (45.0%)	0.800	0.780	0.900	0.940
	Optimization	**428.9–3745.0**^a^	**113 (58.2%)**	**43 (56.6%)**	**11 (55.0%)**	(0.730)	(0.520)	(0.700)	(0.810)
	DFS-Based	1.6–549.1	114 (58.8%)	48 (63.2%)	9 (45.0%)	0.510	0.240	0.150	0.340
	Optimization	**549.1–3745.0**^a^	**80 (41.2%)**	**28 (36.8%)**	**11 (55.0%)**	(0.350)	(0.480)	(0.210)	(0.410)
						
CXCL-9 (MIG, pg/ml)	Median	–	274	154	439	0.190	0.190	0.070	0.140
	(25th–75th)		(119–504)	(83–540)	(142–644)	(0.100)	(0.730)	(0.270)	(0.240)
	Quartiles	1.9–103.9	46 (23.7%)	23 (30.3%)	3 (15.0%)	0.120	0.670	0.240	0.240
		104.9–263.1	47 (24.2%)	22 (28.9%)	4 (20.0%)				
		264.3–512.2	55 (28.4%)	11 (14.5%)	6 (30.0%)				
		519.4–2691.0	46 (23.7%)	20 (26.3%)	7 (35.0%)				
	OS-Based	1.9–120.1	49 (25.3%)	30 (39.5%)	3 (15.0%)	0.022	0.420	0.051	0.026
	Optimization	**121.4–2691.0**^a^	**145 (74.7%)**	**46 (60.5%)**	**17 (85.0%)**	(0.005)	(0.550)	(0.070)	(0.012)
	DFS-Based	1.9–120.1	49 (25.3%)	30 (39.5%)	3 (15.0%)	0.022	0.420	0.051	0.026
	Optimization	**121.4–2691.0**^a^	**145 (74.7%)**	**46 (60.5%)**	**17 (85.0%)**	(0.005)	(0.550)	(0.070)	(0.012)
									
CXCL-8 (IL-8, pg/ml)	Median	–	4.44	6.02	5.83	0.001	0.035	0.880	0.002
	(25th–75th)		(2.50–6.86)	(4.26–8.72)	(4.57–8.80)	(0.060)	(0.180)	(0.880)	(0.120)
	Quartiles	0.36–3.07	61 (31.4%)	10 (13.2%)	3 (15.0%)	0.005	0.180	0.350	0.009
		3.15–4.89	47 (24.2%)	22 (28.9%)	3 (15.0%)				
		4.91–7.53	47 (24.2%)	17 (22.4%)	8 (40.0%)				
		7.68–74.69	39 (20.1%)	27 (35.5%)	6 (30.0%)				
	OS-Based	0.36–17.15	187 (96.4%)	74 (97.4%)	19 (95.0%)	1.000	0.550	0.510	0.750
	Optimization	**19.66–74.69**^a^	**7 (3.6%)**	**2 (2.6%)**	**1 (5.0%)**	(0.640)	(0.570)	(0.330)	(0.830)
	DFS-Based	0.36–17.15	187 (96.4%)	74 (97.4%)	19 (95.0%)	1.000	0.550	0.510	0.750
	Optimization	**19.66–74.69**^a^	**7 (3.6%)**	**2 (2.6%)**	**1 (5.0%)**	(0.640)	(0.570)	(0.330)	(0.830)
									
IL-5 (pg/ml)	Median, ng/ml	–	0.48	0.45	0.45	0.039	0.730	0.570	0.130
	(25th–75th)		(0.35–0.77)	(0.3–0.77)	(0.3–0.76)	(0.390)	(0.330)	(0.130)	(0.270)
	Quartiles	0.08–0.30	38 (19.6%)	30 (39.5%)	7 (35.0%)	0.005	0.230	0.910	0.022
		0.35–0.48	76 (39.2%)	18 (23.7%)	4 (20.0%)				
		0.57–0.77	49 (25.3%)	16 (21.1%)	5 (25.0%)				
		0.85–118	31 (16.0%)	12 (15.8%)	4 (20.0%)				
	OS-Based	0.08–0.38	66 (34.0%)	37 (48.7%)	8 (40.0%)	0.027	0.590	0.490	0.080
	Optimization	**0.45–118.0**^a^	**128 (66.0%)**	**39 (51.3%)**	**12 (60.0%)**	(0.090)	(0.660)	(0.630)	(0.240)
	DFS-Based	0.08–0.38	66 (34.0%)	37 (48.7%)	8 (40.0%)	0.027	0.590	0.490	0.080
	Optimization	**0.45–118.0**^a^	**128 (66.0%)**	**39 (51.3%)**	**12 (60.0%)**	(0.090)	(0.660)	(0.630)	(0.240)
									
IL-10 (pg/ml)	Median, ng/ml	–	1.60	1.95	3.23	0.680	0.130	0.330	0.350
	(25th–75th)		(1.60–6.59)	(1.60–9.31)	(2.67–9.13)	(0.500)	(0.500)	(0.790)	(0.610)
	Quartiles	0.18–1.6	100 (51.5%)	35 (46.1%)	5 (25.0%)	0.750	0.050	0.130	0.160
		1.61–1.78	3 (1.5%)	2 (2.6%)	0 (0%)				
		1.82–8.80	44 (22.7%)	18 (23.7%)	10 (50.0%)				
		8.96–1197.53	47 (24.2%)	21 (27.6%)	5 (25.0%)				
	OS-Based	0.18–3.20	114 (58.8%)	41 (53.9%)	7 (35.0%)	0.470	0.047	0.140	0.120
	Optimization	**3.20–1197.53**^a^	**80 (41.2%)**	**35 (46.1%)**	**13 (65.0%)**	(0.320)	(0.048)	(0.130)	(0.100)
	DFS-Based	0.18–1.90	105 (54.1%)	38 (50.0%)	5 (25.0%)	0.540	0.018	0.052	0.045
	Optimization	**2.00–1197.53**^a^	**89 (45.9%)**	**38 (50.0%)**	**15 (75.0%)**	(0.400)	(0.013)	(0.044)	(0.039)
						
IL-13 (pg/ml)	Median, ng/ml	–	1.60	1.60	1.60	0.430	0.770	0.750	0.710
	(25th–75th)		(1.60–4.49)	(1.60–4.31)	(1.60–3.00)	(0.540)	(0.680)	(0.590)	(0.730)
	OS-Based	0.00–1.55	24 (12.4%)	13 (17.1%)	2 (10.0%)	0.310	1.000	0.730	0.560
	Optimization	**1.60–1239.25**^a^	**170 (87.6%)**	**63 (82.9%)**	**18 (90.0%)**	(0.230)	(0.850)	(0.770)	(0.450)
	DFS-Based	0.00–1.01	19 (9.8%)	10 (13.2%)	0 (0%)	0.420	0.230	0.110	0.220
	Optimization	**1.16–1239.25**^a^	**175 (90.2%)**	**66 (86.8%)**	**20 (100%)**	(0.350)	(0.039)	(0.060)	(0.090)

a Overall survival (OS)- and disease-free survival (DFS)-optimized biomarker ranges associated with poorer outcomes are represented in bold. ALQ=above limit of quantitation. Unadjusted p-values: p^1^, compares *no insulin versus control*; p^2^, compares *any insulin versus control*; p^3^, compares *any insulin versus no insulin* (as per Kruskal-Wallis test); global test, compares *all categories* (as per Wilcoxon, type 3 error test); MVP, denotes the p-value of each multivariate adjusted analysis corresponding to the earlier described unadjusted analyses. For more information, please see Section 2.7 below and our previously published analysis work flow [1]. Interleukine-2, IL-2; soluble interleukine-2 receptor α, sIL-2Rα interleukine-12 subunit p40, IL-12p40; interleukine-12 subunit p70, IL-12p70; interferon α 2, IFN-α2; interferon γ, IFN-γ chemokine ligand 10, CXCL-10 (interferon gamma-induced protein 10, IP-10); chemokine ligand 9, CXCL-9 (monokine-induced by interferon γ, MIG); chemokine ligand 8, CXCL-8 (interleukine-8, IL-8); interleukine-5, IL-5; interleukine-10, IL-10; interleukine-13, IL-13.

**Table 2:** T-helper 1 and 2 produced cytokines׳ correlations by insulin use.

Compared Biomarkers		Group	Unadjusted Correlation			Adjusted Correlation		
			Pearson Correlation	95% Confidence Interval	p-value	Pearson Correlation	95% Confidence Interval	p-value
IL-2	sIL-2Rα	All Subjects (*n*=291)	**0.268**	**0.158 to 0.371**	**<0.001**	**0.278**	**0.168 to 0.381**	**<0.001**
		Controls (*n*=194)	**0.197**	**0.058 to 0.329**	**0.006**	**0.212**	**0.072 to 0.344**	**0.003**
		No Insulin (*n*=77)	**0.648**	**0.496 to 0.761**	**<0.001**	**0.649**	**0.494 to 0.764**	**<0.001**
		Any Insulin (*n*=20)	0.001	−0.442 to 0.443	0.996	−0.075	−0.537 to 0.421	0.771
								
IL-2	IL-12p40	All Subjects (*n*=291)	**0.454**	**0.357 to 0.540**	**<0.001**	**0.454**	**0.357 to 0.541**	**<0.001**
		Controls (*n*=194)	**0.711**	**0.634 to 0.775**	**<0.001**	**0.722**	**0.647 to 0.784**	**<0.001**
		No Insulin (*n*=77)	**0.406**	**0.200 to 0.577**	**<0.001**	**0.416**	**0.207 to 0.588**	**<0.001**
		Any Insulin (*n*=20)	**0.871**	**0.696 to 0.948**	**<0.001**	**0.879**	**0.689 to 0.956**	**<0.001**
								
IL-2	IL-12p70	All Subjects (*n*=291)	**0.250**	**0.139 to 0.354**	**<0.001**	**0.253**	**0.142 to 0.358**	**<0.001**
		Controls (*n*=194)	**0.461**	**0.342 to 0.565**	**<0.001**	**0.463**	**0.344 to 0.568**	**<0.001**
		No Insulin (*n*=77)	**0.224**	**0.000 to 0.427**	**0.048**	**0.233**	**0.004 to 0.438**	**0.044**
		Any Insulin (*n*=20)	**0.994**	**0.984 to 0.998**	**<0.001**	**0.995**	**0.986 to 0.998**	**<0.001**
								
IL-2	IFN-α2	All Subjects (*n*=291)	**0.339**	**0.233 to 0.437**	**<0.001**	**0.339**	**0.232 to 0.437**	**<0.001**
		Controls (*n*=194)	**0.494**	**0.380 to 0.594**	**<0.001**	**0.493**	**0.378 to 0.594**	**<0.001**
		No Insulin (*n*=77)	**0.523**	**0.339 to 0.669**	**<0.001**	**0.530**	**0.343 to 0.676**	**<0.001**
		Any Insulin (*n*=20)	**0.975**	**0.936 to 0.990**	**<0.001**	**0.983**	**0.951 to 0.994**	**<0.001**
								
IL-2	IFN-γ	All Subjects (*n*=291)	**0.379**	**0.276 to 0.473**	**<0.001**	**0.387**	**0.285 to 0.481**	**<0.001**
		Controls (*n*=194)	**0.529**	**0.419 to 0.623**	**<0.001**	**0.531**	**0.421 to 0.626**	**<0.001**
		No Insulin (*n*=77)	**0.511**	**0.324 to 0.659**	**<0.001**	**0.517**	**0.327 to 0.667**	**<0.001**
		Any Insulin (*n*=20)	**0.841**	**0.635 to 0.935**	**<0.001**	**0.871**	**0.671 to 0.953**	**<0.001**
								
IL-2	CXCL-10 (IP-10)	All Subjects (*n*=291)	−0.027	−0.142 to 0.088	0.641	−0.031	−0.146 to 0.085	0.603
		Controls (*n*=194)	0.011	−0.130 to 0.152	0.874	0.009	−0.130–0.151	0.898
		No Insulin (*n*=77)	−0.079	−0.297 to 0.148	0.495	−0.079	−0.302–0.152	0.502
		Any Insulin (*n*=20)	0.104	−0.355 to 0.522	0.660	0.179	−0.330–0.608	0.484
								
IL-2	CXCL-9 (MIG)	All Subjects (*n*=291)	**0.192**	**0.079 to 0.300**	**0.001**	**0.183**	**0.069 to 0.293**	**0.002**
		Controls (*n*=194)	**0.170**	**0.030 to 0.303**	**0.018**	**0.160**	**0.018 to 0.295**	**0.027**
		No Insulin (*n*=77)	0.053	−0.173 to 0.273	0.649	0.056	−0.175 to 0.280	0.637
		Any Insulin (*n*=20)	**0.662**	**0.310 to 0.854**	**<0.001**	**0.625**	**0.207 to 0.850**	**0.005**
								
IL-2	CXCL-8 (IL-8)	All Subjects (*n*=291)	**0.163**	**0.049 to 0.273**	**0.005**	**0.159**	**0.044 to 0.270**	**0.007**
		Controls (*n*=194)	**0.379**	**0.252 to 0.494**	**<0.001**	**0.396**	**0.269 to 0.509**	**<0.001**
		No Insulin (*n*=77)	0.090	−0.136 to 0.308	0.432	0.076	−0.155 to 0.299	0.519
		Any Insulin (*n*=20)	**0.797**	**0.548 to 0.916**	**<0.001**	**0.801**	**0.521 to 0.925**	**<0.001**
								
IL-2	IL-5	All Subjects (*n*=291)	0.082	−0.034 to 0.195	0.164	0.080	−0.036 to 0.193	0.177
		Controls (*n*=194)	0.060	−0.082 to 0.199	0.406	0.057	−0.086 to 0.197	0.433
		No Insulin (*n*=77)	0.147	−0.079 to 0.359	0.199	0.156	−0.075 to 0.371	0.182
		Any Insulin (*n*=20)	**0.554**	**0.148 to 0.801**	**0.008**	**0.517**	**0.048 to 0.799**	**0.028**
								
IL-2	IL-10	All Subjects (*n*=291)	**0.174**	**0.060 to 0.283**	**0.003**	**0.180**	**0.066 to 0.289**	**0.002**
		Controls (*n*=194)	**0.553**	**0.447 to 0.644**	**<0.001**	**0.565**	**0.460 to 0.654**	**<0.001**
		No Insulin (*n*=77)	0.149	−0.078 to 0.361	0.194	0.155	−0.077 to 0.370	0.186
		Any Insulin (*n*=20)	0.364	−0.094 to 0.694	0.107	0.471	−0.013 to 0.776	0.049
								
IL-2	IL-13	All Subjects (*n*=291)	0.102	−0.013 to 0.214	0.082	0.111	−0.005 to 0.224	0.059
		Controls (*n*=194)	**0.235**	**0.098 to 0.364**	**<0.001**	**0.241**	**0.103 to 0.371**	**<0.001**
		No Insulin (*n*=77)	**0.246**	**0.023 to 0.445**	**0.030**	**0.252**	**0.024 to 0.454**	**0.029**
		Any Insulin (*n*=20)	**0.715**	**0.399 to 0.879**	**<0.001**	**0.788**	**0.495 to 0.920**	**<0.001**
								
sIL-2Rα	IL-12p40	All Subjects (*n*=291)	**0.355**	**0.251 to 0.452**	**<0.001**	**0.357**	**0.252 to 0.454**	**<0.001**
		Controls (*n*=194)	**0.142**	**0.001 to 0.277**	**0.048**	**0.145**	**0.003 to 0.281**	**0.044**
		No Insulin (*n*=77)	**0.770**	**0.660 to 0.848**	**<0.001**	**0.775**	**0.664 to 0.853**	**<0.001**
		Any Insulin (*n*=20)	0.301	−0.163 to 0.656	0.188	0.273	−0.239 to 0.666	0.279
								
sIL-2Rα	IL-12p70	All Subjects (*n*=291)	**0.210**	**0.097 to 0.317**	**<0.001**	**0.208**	**0.095 to 0.316**	**<0.001**
		Controls (*n*=194)	0.009	−0.132 to 0.150	0.896	0.012	−0.130 to 0.154	0.868
		No Insulin (*n*=77)	**0.600**	**0.434 to 0.726**	**<0.001**	**0.606**	**0.438 to 0.733**	**<0.001**
		Any Insulin (*n*=20)	−0.035	−0.470 to 0.414	0.884	−0.122	−0.569 to 0.381	0.636
								
sIL-2Rα	IFN-α2	All Subjects (*n*=291)	**0.164**	**0.050 to 0.274**	**0.005**	**0.165**	**0.050 to 0.275**	**0.005**
		Controls (*n*=194)	0.042	−0.100 to 0.182	0.563	0.046	−0.096 to 0.187	0.526
		No Insulin (*n*=77)	**0.744**	**0.624 to 0.830**	**<0.001**	**0.750**	**0.629 to 0.835**	**<0.001**
		Any Insulin (*n*=20)	0.138	−0.325 to 0.547	0.558	0.028	−0.459 to 0.502	0.914
								
sIL-2Rα	IFN-γ	All Subjects (*n*=291)	**0.468**	**0.373 to 0.553**	**<0.001**	**0.466**	**0.371 to 0.552**	**<0.001**
		Controls (*n*=194)	**0.466**	**0.348 to 0.569**	**<0.001**	**0.469**	**0.350 to 0.572**	**<0.001**
		No Insulin (*n*=77)	**0.629**	**0.471 to 0.747**	**<0.001**	**0.635**	**0.476 to 0.754**	**<0.001**
		Any Insulin (*n*=20)	0.114	−0.346 to 0.530	0.628	0.243	−0.269 to 0.648	0.339
								
sIL-2Rα	CXCL-10 (IP-10)	All Subjects (*n*=291)	−0.039	−0.153 to 0.077	0.511	−0.032	−0.147 to 0.084	0.587
		Controls (*n*=194)	−0.038	−0.178 to 0.104	0.599	−0.027	−0.168 to 0.115	0.709
		No Insulin (*n*=77)	0.014	−0.211 to 0.237	0.905	0.011	−0.218 to 0.239	0.923
		Any Insulin (*n*=20)	−0.213	−0.599 to 0.254	0.361	0.231	−0.281 to 0.640	0.364
								
sIL-2Rα	CXCL-9 (MIG)	All Subjects (*n*=291)	**0.119**	**0.004 to 0.231**	**0.042**	**0.123**	**0.007 to 0.235**	**0.037**
		Controls (*n*=194)	**0.150**	**0.009 to 0.285**	**0.036**	**0.158**	**0.016 to 0.293**	**0.029**
		No Insulin (*n*=77)	0.073	−0.154 to 0.292	0.528	0.075	−0.156 to 0.299	0.521
		Any Insulin (*n*=20)	0.021	−0.426 to 0.459	0.931	−0.145	−0.585 to 0.360	0.572
								
sIL-2Rα	CXCL-8 (IL-8)	All Subjects (*n*=291)	**0.146**	**0.032 to 0.257**	**0.012**	**0.155**	**0.040 to 0.266**	**0.008**
		Controls (*n*=194)	**0.149**	**0.008 to 0.284**	**0.038**	**0.150**	**0.008 to 0.286**	**0.037**
		No Insulin (*n*=77)	**0.298**	**0.079 to 0.489**	**0.008**	**0.301**	**0.077 to 0.495**	**0.008**
		Any Insulin (*n*=20)	−0.122	−0.536 to 0.339	0.604	−0.283	−0.672 to 0.229	0.262
								
sIL-2Rα	IL-5	All Subjects (*n*=291)	0.091	−0.024 to 0.204	0.121	0.092	−0.023 to 0.206	0.117
		Controls (*n*=194)	−0.023	−0.163 to 0.118	0.752	−0.020	−0.161 to 0.123	0.788
		No Insulin (*n*=77)	**0.619**	**0.459 to 0.741**	**<0.001**	**0.625**	**0.463 to 0.747**	**<0.001**
		Any Insulin (*n*=20)	**0.560**	**0.156 to 0.803**	**0.008**	**0.562**	**0.112 to 0.821**	**0.014**
								
sIL-2Rα	IL-10	All Subjects (*n*=291)	**0.236**	**0.124 to 0.341**	**<0.001**	**0.234**	**0.122 to 0.340**	**<0.001**
		Controls (*n*=194)	0.054	−0.088 to 0.193	0.456	0.053	−0.090 to 0.193	0.470
		No Insulin (*n*=77)	**0.605**	**0.440 to 0.730**	**<0.001**	**0.608**	**0.441 to 0.734**	**<0.001**
		Any Insulin (*n*=20)	−0.101	−0.520 to 0.357	0.668	0.282	−0.229 to 0.672	0.263
								
sIL-2Rα	IL-13	All Subjects (*n*=291)	0.050	−0.065 to 0.164	0.391	0.046	−0.070 to 0.161	0.433
		Controls (*n*=194)	−0.014	−0.155 to 0.127	0.841	−0.019	−0.161 to 0.123	0.792
		No Insulin (*n*=77)	**0.555**	**0.378 to 0.693**	**<0.001**	**0.558**	**0.377 to 0.697**	**<0.001**
		Any Insulin (*n*=20)	−0.169	−0.568 to 0.297	0.473	−0.061	−0.526 to 0.432	0.814
								
IL-12p40	IL-12p70	All Subjects (*n*=291)	**0.853**	**0.819 to 0.882**	**<0.001**	**0.854**	**0.819 to 0.883**	**<0.001**
		Controls (*n*=194)	**0.653**	**0.564 to 0.727**	**<0.001**	**0.655**	**0.565 to 0.729**	**<0.001**
		No Insulin (*n*=77)	**0.932**	**0.895 to 0.956**	**<0.001**	**0.931**	**0.893 to 0.956**	**<0.001**
		Any Insulin (*n*=20)	**0.825**	**0.602 to 0.928**	**<0.001**	**0.842**	**0.607 to 0.942**	**<0.001**
								
IL-12p40	IFN-α2	All Subjects (*n*=291)	**0.591**	**0.510 to 0.661**	**<0.001**	**0.590**	**0.510 to 0.661**	**<0.001**
		Controls (*n*=194)	**0.721**	**0.645 to 0.782**	**<0.001**	**0.725**	**0.649 to 0.786**	**<0.001**
		No Insulin (*n*=77)	**0.891**	**0.834 to 0.930**	**<0.001**	**0.892**	**0.834 to 0.931**	**<0.001**
		Any Insulin (*n*=20)	**0.940**	**0.852 to 0.976**	**<0.001**	**0.935**	**0.825 to 0.977**	**<0.001**
								
IL-12p40	IFN-γ	All Subjects (*n*=291)	**0.492**	**0.399 to 0.574**	**<0.001**	**0.493**	**0.400 to 0.575**	**<0.001**
		Controls (*n*=194)	**0.473**	**0.356 to 0.576**	**<0.001**	**0.482**	**0.365 to 0.584**	**<0.001**
		No Insulin (*n*=77)	**0.662**	**0.515 to 0.772**	**<0.001**	**0.665**	**0.515 to 0.776**	**<0.001**
		Any Insulin (*n*=20)	**0.736**	**0.436 to 0.889**	**<0.001**	**0.818**	**0.557 to 0.932**	**<0.001**
								
IL-12p40	CXCL-10 (IP-10)	All Subjects (*n*=291)	0.041	−0.074 to 0.155	0.484	0.044	−0.72 to 0.159	0.458
		Controls (*n*=194)	0.082	−0.059 to 0.221	0.252	0.077	−0.066 to 0.217	0.290
		No Insulin (*n*=77)	0.060	−0.166 to 0.280	0.604	0.058	−0.173 to 0.282	0.625
		Any Insulin (*n*=20)	0.040	−0.410 to 0.474	0.865	0.180	−0.329 to 0.608	0.481
								
IL-12p40	CXCL-9 (MIG)	All Subjects (*n*=291)	**0.172**	**0.058 to 0.281**	**0.003**	**0.169**	**0.054 to 0.280**	**0.004**
		Controls (*n*=194)	**0.248**	**0.111 to 0.376**	**<0.001**	**0.249**	**0.111 to 0.378**	**<0.001**
		No Insulin (*n*=77)	0.132	−0.095 to 0.246	0.250	0.134	−0.098 to 0.351	0.254
		Any Insulin (*n*=20)	**0.543**	**0.132 to 0.794**	**0.010**	0.433	−0.060 to 0.756	0.074
								
IL-12p40	CXCL-8 (IL-8)	All Subjects (*n*=291)	**0.292**	**0.183 to 0.394**	**<0.001**	**0.295**	**0.186 to 0.397**	**<0.001**
		Controls (*n*=194)	**0.571**	**0.467 to 0.659**	**<0.001**	**0.572**	**0.468 to 0.660**	**<0.001**
		No Insulin (*n*=77)	**0.240**	**0.017 to 0.441**	**0.034**	**0.264**	**0.038 to 0.465**	**0.022**
		Any Insulin (*n*=20)	**0.650**	**0.291 to 0.848**	**0.001**	**0.551**	**0.096 to 0.816**	**0.017**
								
IL-12p40	IL-5	All Subjects (*n*=291)	**0.297**	**0.188 to 0.398**	**<0.001**	**0.296**	**0.187 to 0.398**	**<0.001**
		Controls (*n*=194)	0.070	−0.071 to 0.209	0.329	0.071	−0.072 to 0.211	0.328
		No Insulin (*n*=77)	**0.948**	**0.919 to 0.967**	**<0.001**	**0.947**	**0.918 to 0.967**	**<0.001**
		Any Insulin (*n*=20)	**0.854**	**0.661 to 0.941**	**<0.001**	**0.817**	**0.555 to 0.932**	**<0.001**
								
IL-12p40	IL-10	All Subjects (*n*=291)	**0.909**	**0.886 to 0.927**	**<0.001**	**0.910**	**0.888 to 0.928**	**<0.001**
		Controls (*n*=194)	**0.904**	**0.874 to 0.927**	**<0.001**	**0.905**	**0.875 to 0.928**	**<0.001**
		No Insulin (*n*=77)	**0.943**	**0.911 to 0.963**	**<0.001**	**0.942**	**0.909 to 0.963**	**<0.001**
		Any Insulin (*n*=20)	0.331	−0.131 to 0.674	0.146	**0.492**	**0.015 to 0.787**	**0.038**
								
IL-12p40	IL-13	All Subjects (*n*=291)	**0.374**	**0.271 to 0.469**	**<0.001**	**0.376**	**0.273 to 0.471**	**<0.001**
		Controls (*n*=194)	**0.444**	**0.323 to 0.550**	**<0.001**	**0.449**	**0.327 to 0.555**	**<0.001**
		No Insulin (*n*=77)	**0.881**	**0.818 to 0.923**	**<0.001**	**0.880**	**0.815 to 0.923**	**<0.001**
		Any Insulin (*n*=20)	**0.563**	**0.161 to 0.805**	**0.007**	**0.685**	**0.304 to 0.877**	**0.001**
								
IL-12p70	IFN-α2	All Subjects (*n*=291)	**0.749**	**0.693 to 0.795**	**<0.001**	**0.749**	**0.693 to 0.796**	**<0.001**
		Controls (*n*=194)	**0.944**	**0.926 to 0.958**	**<0.001**	**0.944**	**0.926 to 0.958**	**<0.001**
		No Insulin (*n*=77)	**0.839**	**0.757 to 0.895**	**<0.001**	**0.836**	**0.751 to 0.894**	**<0.001**
		Any Insulin (*n*=20)	**0.959**	**0.898 to 0.984**	**<0.001**	**0.972**	**0.922 to 0.990**	**<0.001**
								
IL-12p70	IFN-γ	All Subjects (*n*=291)	**0.526**	**0.438 to 0.605**	**<0.001**	**0.526**	**0.437 to 0.605**	**<0.001**
		Controls (*n*=194)	**0.506**	**0.393 to 0.604**	**<0.001**	**0.508**	**0.394 to 0.606**	**<0.001**
		No Insulin (*n*=77)	**0.641**	**0.488 to 0.757**	**<0.001**	**0.638**	**0.479 to 0.756**	**<0.001**
		Any Insulin (*n*=20)	**0.831**	**0.615 to 0.931**	**<0.001**	**0.860**	**0.646 to 0.948**	**<0.001**
								
IL-12p70	CXCL-10 (IP-10)	All Subjects (*n*=291)	0.047	−0.069 to 0.161	0.426	0.053	−0.063 to 0.168	0.367
		Controls (*n*=194)	0.059	−0.082 to 0.199	0.411	0.063	−0.080 to 0.203	0.386
		No Insulin (*n*=77)	0.073	−0.153 to 0.292	0.525	0.071	−0.160 to 0.295	0.547
		Any Insulin (*n*=20)	0.098	−0.360 to 0.518	0.678	0.183	−0.326 to 0.610	0.474
								
IL-12p70	CXCL-9 (MIG)	All Subjects (*n*=291)	**0.235**	**0.124 to 0.341**	**<0.001**	**0.235**	**0.123 to 0.342**	**<0.001**
		Controls (*n*=194)	**0.377**	**0.249 to 0.492**	**<0.001**	**0.371**	**0.242 to 0.487**	**<0.001**
		No Insulin (*n*=77)	0.180	−0.046 to 0.389	0.115	0.184	−0.046 to 0.396	0.114
		Any Insulin (*n*=20)	**0.644**	**0.281 to 0.845**	**0.001**	**0.612**	**0.186 to 0.844**	**0.006**
								
IL-12p70	CXCL-8 (IL-8)	All Subjects (*n*=291)	**0.182**	**0.069 to 0.291**	**0.002**	**0.188**	**0.074 to 0.297**	**<0.001**
		Controls (*n*=194)	**0.203**	**0.064 to 0.335**	**0.004**	**0.210**	**0.070 to 0.342**	**0.003**
		No Insulin (*n*=77)	**0.249**	**0.026 to 0.448**	**0.028**	**0.278**	**0.052 to 0.476**	**0.016**
		Any Insulin (*n*=20)	**0.812**	**0.576 to 0.923**	**<0.001**	**0.832**	**0.585 to 0.938**	**<0.001**
								
IL-12p70	IL-5	All Subjects (*n*=291)	**0.254**	**0.143 to 0.358**	**<0.001**	**0.255**	**0.143 to 0.360**	**<0.001**
		Controls (*n*=194)	0.030	−0.111 to 0.171	0.674	0.033	−0.109 to 0.174	0.649
		No Insulin (*n*=77)	**0.969**	**0.952 to 0.980**	**<0.001**	**0.969**	**0.951 to 0.980**	**<0.001**
		Any Insulin (*n*=20)	**0.495**	**0.067 to 0.769**	**0.022**	0.465	−0.021 to 0.773	0.052
								
IL-12p70	IL-10	All Subjects (*n*=291)	**0.897**	**0.872 to 0.917**	**<0.001**	**0.897**	**0.872 to 0.918**	**<0.001**
		Controls (*n*=194)	**0.709**	**0.631 to 0.773**	**<0.001**	**0.709**	**0.630 to 0.773**	**<0.001**
		No Insulin (*n*=77)	**0.973**	**0.957 to 0.982**	**<0.001**	**0.973**	**0.957 to 0.983**	**<0.001**
		Any Insulin (*n*=20)	0.336	−0.126 to 0.678	0.140	0.447	−0.042 to 0.764	0.064
								
IL-12p70	IL-13	All Subjects (*n*=291)	**0.412**	**0.312 to 0.503**	**<0.001**	**0.413**	**0.312 to 0.504**	**<0.001**
		Controls (*n*=194)	**0.375**	**0.247 to 0.490**	**<0.001**	**0.380**	**0.252 to 0.495**	**<0.001**
		No Insulin (*n*=77)	**0.961**	**0.939 to 0.975**	**<0.001**	**0.960**	**0.938 to 0.975**	**<0.001**
		Any Insulin (*n*=20)	**0.724**	**0.415 to 0.884**	**<0.001**	**0.794**	**0.506 to 0.923**	**<0.001**
								
IFN-α2	IFN-γ	All Subjects (*n*=291)	**0.620**	**0.544 to 0.686**	**<0.001**	**0.622**	**0.546 to 0.688**	**<0.001**
		Controls (*n*=194)	**0.571**	**0.468 to 0.659**	**<0.001**	**0.571**	**0.467 to 0.660**	**<0.001**
		No Insulin (*n*=77)	**0.810**	**0.716 to 0.875**	**<0.001**	**0.807**	**0.709 to 0.874**	**<0.001**
		Any Insulin (*n*=20)	**0.793**	**0.539 to 0.914**	**<0.001**	**0.862**	**0.651 to 0.949**	**<0.001**
								
IFN-α2	CXCL-10 (IP-10)	All Subjects (*n*=291)	0.047	−0.068 to 0.161	0.424	0.053	−0.063 to 0.167	0.370
		Controls (*n*=194)	0.056	−0.086 to 0.195	0.440	0.0616	−0.081 to 0.202	0.396
		No Insulin (*n*=77)	0.003	−0.221 to 0.227	0.978	0.000	−0.228 to 0.229	0.999
		Any Insulin (*n*=20)	0.062	−0.391 to 0.491	0.793	0.230	−0.282 to 0.640	0.366
								
IFN-α2	CXCL-9 (MIG)	All Subjects (*n*=291)	**0.345**	**0.240 to 0.443**	**<0.001**	**0.342**	**0.236 to 0.441**	**<0.001**
		Controls (*n*=194)	**0.413**	**0.289 to 0.524**	**<0.001**	**0.406**	**0.280 to 0.518**	**<0.001**
		No Insulin (*n*=77)	0.106	−0.120 to 0.323	0.355	0.110	−0.121 to 0.331	0.347
		Any Insulin (*n*=20)	**0.623**	**0.250 to 0.835**	**0.002**	**0.546**	**0.089 to 0.813**	**0.018**
								
IFN-α2	CXCL-8 (IL-8)	All Subjects (*n*=291)	**0.397**	**0.296 to 0.490**	**<0.001**	**0.403**	**0.302 to 0.496**	**<0.001**
		Controls (*n*=194)	**0.396**	**0.270 to 0.508**	**<0.001**	**0.409**	**0.283 to 0.521**	**<0.001**
		No Insulin (*n*=77)	**0.315**	**0.097 to 0.503**	**0.005**	**0.331**	**0.111 to 0.520**	**0.004**
		Any Insulin (*n*=20)	**0.768**	**0.494 to 0.904**	**<0.001**	**0.735**	**0.394 to 0.898**	**<0.001**
								
IFN-α2	IL-5	All Subjects (*n*=291)	**0.146**	**0.032 to 0.257**	**0.012**	**0.147**	**0.032 to 0.258**	**0.012**
		Controls (*n*=194)	0.043	−0.099 to 0.182	0.554	0.045	−0.097 to 0.186	0.533
		No Insulin (*n*=77)	**0.792**	**0.690 to 0.863**	**<0.001**	**0.790**	**0.685 to 0.863**	**<0.001**
		Any Insulin (*n*=20)	**0.703**	**0.378 to 0.874**	**<0.001**	**0.634**	**0.220 to 0.854**	**0.004**
								
IFN-α2	IL-10	All Subjects (*n*=291)	**0.655**	**0.584 to 0.716**	**<0.001**	**0.657**	**0.586 to 0.718**	**<0.001**
		Controls (*n*=194)	**0.813**	**0.758 to 0.855**	**<0.001**	**0.480**	**0.362 to 0.582**	**<0.001**
		No Insulin (*n*=77)	**0.823**	**0.734 to 0.884**	**<0.001**	**0.821**	**0.729 to 0.884**	**<0.001**
		Any Insulin (*n*=20)	0.279	−0.187 to 0.642	0.226	0.435	−0.057 to 0.758	0.072
								
IFN-α2	IL-13	All Subjects (*n*=291)	**0.556**	**0.471 to 0.630**	**<0.001**	**0.560**	**0.475 to 0.635**	**<0.001**
		Controls (*n*=194)	**0.538**	**0.429 to 0.631**	**<0.001**	**0.545**	**0.437 to 0.637**	**<0.001**
		No Insulin (*n*=77)	**0.807**	**0.712 to 0.873**	**<0.001**	**0.802**	**0.703 to 0.871**	**<0.001**
		Any Insulin (*n*=20)	**0.659**	**0.305 to 0.853**	**<0.001**	**0.778**	**0.475 to 0.916**	**<0.001**
								
IFN-γ	CXCL-10 (IP-10)	All Subjects (*n*=291)	0.062	−0.054 to 0.175	0.295	0.068	−0.048 to 0.182	0.251
		Controls (*n*=194)	0.085	−0.057 to 0.223	0.239	0.103	−0.040 to 0.241	0.156
		No Insulin (*n*=77)	−0.055	−0.275 to 0.172	0.636	−0.062	−0.287 to 0.169	0.598
		Any Insulin (*n*=20)	0.340	−0.121 to 0.680	0.134	0.278	−0.234 to 0.669	0.271
								
IFN-γ	CXCL-9 (MIG)	All Subjects (*n*=291)	**0.287**	**0.178 to 0.389**	**<0.001**	**0.291**	**0.181 to 0.393**	**<0.001**
		Controls (*n*=194)	**0.358**	**0.228 to 0.475**	**<0.001**	**0.354**	**0.224 to 0.473**	**<0.001**
		No Insulin (*n*=77)	0.144	−0.082 to 0.357	0.208	0.155	−0.076 to 0.371	0.184
		Any Insulin (*n*=20)	**0.521**	**0.102 to 0.783**	**0.015**	**0.559**	**0.107 to 0.819**	**0.015**
								
IFN-γ	CXCL-8 (IL-8)	All Subjects (*n*=291)	**0.432**	**0.334 to 0.521**	**<0.001**	**0.442**	**0.344 to 0.530**	**<0.001**
		Controls (*n*=194)	**0.485**	**0.370 to 0.586**	**<0.001**	**0.504**	**0.390 to 0.603**	**<0.001**
		No Insulin (*n*=77)	**0.236**	**0.013 to 0.437**	**0.037**	**0.239**	**0.011 to 0.443**	**0.039**
		Any Insulin (*n*=20)	**0.623**	**0.250 to 0.835**	**0.002**	**0.666**	**0.272 to 0.868**	**0.002**
								
IFN-γ	IL-5	All Subjects (*n*=291)	**0.136**	**0.022 to 0.247**	**0.020**	**0.136**	**0.021 to 0.248**	**0.021**
		Controls (*n*=194)	0.047	−0.094 to 0.188	0.514	0.049	−0.093 to 0.190	0.497
		No Insulin (*n*=77)	**0.574**	**0.401 to 0.707**	**<0.001**	**0.570**	**0.393 to 0.707**	**<0.001**
		Any Insulin (*n*=20)	0.421	−0.026 to 0.728	0.058	**0.545**	**0.088 to 0.813**	**0.018**
								
IFN-γ	IL-10	All Subjects (*n*=291)	**0.477**	**0.383 to 0.561**	**<0.001**	**0.476**	**0.382 to 0.561**	**<0.001**
		Controls (*n*=194)	**0.475**	**0.358 to 0.577**	**<0.001**	**0.480**	**0.362 to 0.582**	**<0.001**
		No Insulin (*n*=77)	**0.585**	**0.415 to 0.715**	**<0.001**	**0.583**	**0.409 to 0.716**	**<0.001**
		Any Insulin (*n*=20)	**0.654**	**0.297 to 0.850**	**0.001**	**0.668**	**0.276 to 0.869**	**0.002**
								
IFN-γ	IL-13	All Subjects (*n*=291)	**0.492**	**0.400 to 0.575**	**<0.001**	**0.490**	**0.397 to 0.573**	**<0.001**
		Controls (*n*=194)	**0.503**	**0.390 to 0.601**	**<0.001**	**0.504**	**0.389 to 0.603**	**<0.001**
		No Insulin (*n*=77)	**0.601**	**0.436 to 0.727**	**<0.001**	**0.591**	**0.419 to 0.722**	**<0.001**
		Any Insulin (*n*=20)	**0.849**	**0.650 to 0.939**	**<0.001**	**0.852**	**0.628 to 0.945**	**<0.001**
								
CXCL-10 (IP-10)	CXCL-9 (MIG)	All Subjects (*n*=291)	0.093	−0.022 to 0.206	0.114	0.102	−0.014 to 0.215	0.084
		Controls (*n*=194)	0.089	−0.052 to 0.227	0.216	0.097	−0.046 to 0.235	0.183
		No Insulin (*n*=77)	**0.258**	**0.036 to 0.456**	**0.022**	**0.261**	**0.034 to 0.462**	**0.024**
		Any Insulin (*n*=20)	−0.090	−0.512 to 0.367	0.703	0.061	−0.432 to 0.526	0.813
								
CXCL-10 (IP-10)	CXCL-8 (IL-8)	All Subjects (*n*=291)	0.108	−0.007 to 0.220	0.065	0.095	−0.021 to 0.208	0.108
		Controls (*n*=194)	0.121	−0.020 to 0.258	0.092	0.110	−0.032 to 0.248	0.129
		No Insulin (*n*=77)	0.096	−0.131 to 0.313	0.406	0.098	−0.134 to 0.319	0.406
		Any Insulin (*n*=20)	−0.040	−0.474 to 0.409	0.864	0.059	−0.434 to 0.525	0.818
								
CXCL-10 (IP-10)	IL-5	All Subjects (*n*=291)	0.000	−0.115 to 0.115	0.996	−0.002	−0.117 to 0.114	0.975
		Controls (*n*=194)	−0.007	−0.148 to 0.134	0.918	−0.013	−0.155 to 0.129	0.857
		No Insulin (*n*=77)	0.073	−0.153 to 0.292	0.527	0.070	−0.161 to 0.293	0.554
		Any Insulin (*n*=20)	−0.157	−0.560 to 0.307	0.503	0.129	−0.375 to 0.574	0.616
								
CXCL-10 (IP-10)	IL-10	All Subjects (*n*=291)	0.058	−0.057 to 0.172	0.324	0.061	−0.055 to 0.175	0.302
		Controls (*n*=194)	0.067	−0.075 to 0.206	0.353	0.070	−0.073 to 0.209	0.338
		No Insulin (*n*=77)	0.087	−0.140 to 0.305	0.451	0.085	−0.146 to 0.308	0.470
		Any Insulin (*n*=20)	**0.477**	**0.044 to 0.759**	**0.028**	0.114	−0.388 to 0.564	0.659
								
CXCL-10 (IP-10)	IL-13	All Subjects (*n*=291)	**0.140**	**0.026 to 0.251**	**0.016**	**0.144**	**0.029 to 0.255**	**0.014**
		Controls (*n*=194)	0.140	−0.001 to 0.275	0.051	**0.149**	**0.008 to 0.285**	**0.038**
		No Insulin (*n*=77)	0.119	−0.108 to 0.334	0.302	0.116	−0.115 to 0.336	0.322
		Any Insulin (*n*=20)	0.307	−0.156 to 0.660	0.179	0.205	−0.305 to 0.624	0.421
								
CXCL-9 (MIG)	CXCL-8 (IL-8)	All Subjects (*n*=291)	**0.118**	**0.003 to 0.230**	**0.043**	**0.122**	**0.007 to 0.234**	**0.038**
		Controls (*n*=194)	0.107	−0.035 to 0.244	0.137	0.119	−0.023 to 0.257	0.100
		No Insulin (*n*=77)	−0.022	−0.245 to 0.203	0.849	−0.017	−0.244 to 0.212	0.888
		Any Insulin (*n*=20)	**0.518**	**0.098 to 0.781**	**0.015**	0.426	−0.069 to 0.753	0.079
								
CXCL-9 (MIG)	IL-5	All Subjects (*n*=291)	0.038	−0.077 to 0.153	0.515	0.037	−0.079 to 0.152	0.527
		Controls (*n*=194)	−0.025	−0.165 to 0.117	0.734	−0.023	−0.164 to 0.119	0.752
		No Insulin (*n*=77)	0.162	−0.064 to 0.372	0.157	0.166	−0.065 to 0.380	0.156
		Any Insulin (*n*=20)	0.404	−0.046 to 0.718	0.070	0.192	−0.318 to 0.616	0.453
								
CXCL-9 (MIG)	IL-10	All Subjects (*n*=291)	**0.149**	**0.035 to 0.260**	**0.011**	**0.153**	**0.038 to 0.264**	**0.009**
		Controls (*n*=194)	**0.274**	**0.139 to 0.400**	**<0.001**	**0.274**	**0.137 to 0.400**	**<0.001**
		No Insulin (*n*=77)	0.132	−0.095 to 0.346	0.250	0.134	−0.098 to 0.352	0.253
		Any Insulin (*n*=20)	0.075	−0.380 to 0.501	0.752	0.196	−0.314 to 0.619	0.443
								
CXCL-9 (MIG)	IL-13	All Subjects (*n*=291)	**0.159**	**0.045 to 0.269**	**0.006**	**0.166**	**0.052 to 0.277**	**0.004**
		Controls (*n*=194)	**0.186**	**0.047 to 0.319**	**0.009**	**0.191**	**0.051 to 0.324**	**0.008**
		No Insulin (*n*=77)	0.211	−0.013 to 0.416	0.063	0.217	−0.012 to 0.425	0.061
		Any Insulin (*n*=20)	0.414	−0.035 to 0.724	0.063	0.489	0.011–0.785	0.039
								
CXCL-8 (IL-8)	IL-5	All Subjects (*n*=291)	**0.125**	**0.010 to 0.237**	**0.033**	**0.125**	**0.010 to 0.237**	**0.033**
		Controls (*n*=194)	0.107	−0.034 to 0.245	0.135	0.107	−0.036 to 0.245	0.141
		No Insulin (*n*=77)	0.215	−0.009 to 0.419	0.058	**0.245**	**0.017 to 0.448**	**0.034**
		Any Insulin (*n*=20)	0.424	−0.022 to 0.730	0.056	0.189	−0.321 to 0.614	0.460
								
CXCL-8 (IL-8)	IL-10	All Subjects (*n*=291)	**0.402**	**0.301 to 0.494**	**<0.001**	**0.408**	**0.307 to 0.500**	**<0.001**
		Controls (*n*=194)	**0.672**	**0.586 to 0.742**	**<0.001**	**0.677**	**0.592 to 0.747**	**<0.001**
		No Insulin (*n*=77)	**0.293**	**0.073 to 0.485**	**0.009**	**0.318**	**0.096 to 0.509**	**0.005**
		Any Insulin (*n*=20)	0.260	−0.207 to 0.630	0.261	0.377	−0.127 to 0.726	0.127
								
CXCL-8 (IL-8)	IL-13	All Subjects (*n*=291)	**0.640**	**0.567 to 0.703**	**<0.001**	**0.651**	**0.579 to 0.713**	**<0.001**
		Controls (*n*=194)	**0.687**	**0.605 to 0.755**	**<0.001**	**0.695**	**0.613 to 0.761**	**<0.001**
		No Insulin (*n*=77)	**0.306**	**0.088 to 0.496**	**0.006**	**0.330**	**0.110 to 0.519**	**0.004**
		Any Insulin (*n*=20)	**0.527**	**0.110 to 0.786**	**0.013**	**0.596**	**0.162 to 0.837**	**0.008**
								
IL-5	IL-10	All Subjects (*n*=291)	**0.308**	**0.200 to 0.408**	**<0.001**	**0.309**	**0.200 to 0.410**	**<0.001**
		Controls (*n*=194)	0.134	−0.007 to 0.270	0.270	0.138	−0.004 to 0.275	0.056
		No Insulin (*n*=77)	**0.981**	**0.970 to 0.988**	**<0.001**	**0.981**	**0.970 to 0.988**	**<0.001**
		Any Insulin (*n*=20)	0.033	−0.416 to 0.468	0.890	0.248	−0.264 to 0.651	0.327
								
IL-5	IL-13	All Subjects (*n*=291)	**0.134**	**0.020 to 0.245**	**0.022**	**0.134**	**0.018 to 0.246**	**0.023**
		Controls (*n*=194)	0.065	−0.076 to 0.204	0.364	0.066	−0.077 to 0.206	0.363
		No Insulin (*n*=77)	**0.915**	**0.869 to 0.945**	**<0.001**	**0.914**	**0.866 to 0.945**	**<0.001**
		Any Insulin (*n*=20)	0.233	−0.233 to 0.613	0.314	0.379	−0.125 to 0.727	0.124
								
IL-10	IL-13	All Subjects (*n*=291)	**0.513**	**0.423 to 0.593**	**<0.001**	**0.512**	**0.422 to 0.593**	**<0.001**
		Controls (*n*=194)	**0.596**	**0.497 to 0.680**	**<0.001**	**0.601**	**0.501 to 0.684**	**<0.001**
		No Insulin (*n*=77)	**0.943**	**0.912 to 0.964**	**<0.001**	**0.943**	**0.911 to 0.964**	**<0.001**
		Any Insulin (*n*=20)	**0.508**	**0.084 to 0.776**	**0.018**	0.460	−0.027 to 0.770	0.056

Significant correlations are displayed in bolded text. The differences that are only significant in either adjusted or unadjusted correlations are further denoted by an outline. Interleukine-2, IL-2; soluble interleukine-2 receptor α, sIL-2Rα interleukine-12 subunit p40, IL-12p40; interleukine-12 subunit p70, IL-12p70; interferon α 2, IFN-α2; interferon γ, IFN-γ chemokine ligand 10,CXCL-10 (interferon gamma-induced protein 10, IP-10); chemokine ligand 9, CXCL-9 (monokine-induced by interferon γ, MIG); chemokine ligand 8, CXCL-8 (interleukine-8, IL-8); interleukine-5, IL-5; interleukine-10, IL-10; interleukine-13, IL-13.
